# Cluster-based network modeling—From snapshots to complex dynamical systems

**DOI:** 10.1126/sciadv.abf5006

**Published:** 2021-06-16

**Authors:** Daniel Fernex, Bernd R. Noack, Richard Semaan

**Affiliations:** 1 Institut für Strömungsmechanik, Technische Universität Braunschweig, Hermann-Blenk-Str. 37, 38108 Braunschweig, Germany.; 2 Center for Turbulence Control, Harbin Institute of Technology, Shenzhen 518058, People’s Republic of China.; 3 Hermann-Föttinger-Institut, Technische Universität Berlin, Müller-Breslau-Str. 8, 10623 Berlin, Germany.

## Abstract

We propose a universal method for data-driven modeling of complex nonlinear dynamics from time-resolved snapshot data without prior knowledge. Complex nonlinear dynamics govern many fields of science and engineering. Data-driven dynamic modeling often assumes a low-dimensional subspace or manifold for the state. We liberate ourselves from this assumption by proposing cluster-based network modeling (CNM) bridging machine learning, network science, and statistical physics. CNM describes short- and long-term behavior and is fully automatable, as it does not rely on application-specific knowledge. CNM is demonstrated for the Lorenz attractor, ECG heartbeat signals, Kolmogorov flow, and a high-dimensional actuated turbulent boundary layer. Even the notoriously difficult modeling benchmark of rare events in the Kolmogorov flow is solved. This automatable universal data-driven representation of complex nonlinear dynamics complements and expands network connectivity science and promises new fast-track avenues to understand, estimate, predict, and control complex systems in all scientific fields.

## Teaser

A generally applicable robust data-driven network modeling strategy offers rapid means to predict and control complex systems.

## INTRODUCTION

Climate, epidemiology, brain activity, financial markets, and turbulence constitute examples of complex systems. They are characterized by a large range of time and spatial scales, intrinsic high dimensionality, and nonlinear dynamics. Dynamic modeling for the long-term features is a key enabler for understanding, state estimation from limited sensor signals, prediction, control, and optimization. Data-driven modeling has made tremendous progress in the past decades, driven by algorithmic advances, accessibility to large data, and hardware speedups. Typically, the modeling is based on a low-dimensional approximation of the state and system identification in that approximation.

The low-dimensional approximation may be achieved with subspace modeling methods, such as proper orthogonal decomposition (POD) models (*1*, *2*), dynamic mode decomposition (*3*), and empirical dynamical modeling (*4*), to name only a few. Autoencoders (*5*) represent a general nonlinear dimension reduction to a low-dimensional feature space. The dynamic system identification is substantially simplified in this feature space.

An early breakthrough in system identification was reported by Bongard and Lipson (*6*) using symbolic regression. The method performs a heuristic search of the best equation that describes the dynamics (*7*). They are, however, expensive and not easily scalable to large systems. Recent developments in parsimonious modeling lead to the “sparse identification of nonlinear dynamics” (SINDy) algorithm that identifies accurate parsimonious models from data (*8*). Similarly, SINDy is not easily scalable to large problems. The computational expense becomes exorbitant already for moderate dimensional feature spaces.

This limitation may be bypassed by black box techniques. These include Volterra series (*9*), autoregressive models (e.g., ARX, ARMA, and NARMAX) (*10*), eigensystem realization algorithm (*11*), and neural network (NN) models (*12*). These approaches, however, have limited interpretability and provide little physical insights. Some (e.g., NN) require large volumes of data and long training time, luxuries that are not always at hand.

In this study, we follow an alternative modeling paradigm starting with a time-resolved snapshot set. We liberate ourselves from the requirement of a low-dimensional subspace or manifold for the data and the analytical simplicity assumption of the dynamical system. The snapshots are coarse-grained into a small number of centroids with clustering. The dynamics is described by a network model with continuous transitions between the centroids. The resulting cluster-based network modeling (CNM) uses time-delay embedding to identify models with an arbitrary degree of complexity and nonlinearity. The methodology is developed within the network science (*13*–*15*) and statistical physics (*16*) frameworks. Because of its generic nature, network analysis is being increasingly used to investigate complex systems (*17*, *18*). The proposed method builds on previous work by Kaiser *et al.* (*19*), where clustering is used to coarse-grain the data into representative states and the temporal evolution is modeled as a probabilistic Markov model. By construction, the state vector of cluster probabilities converges to a fixed point representing the posttransient attractor, i.e., the dynamics disappear. A recent improvement (*20*) models the transition dynamics between the network nodes as straight constant-velocity “flights” with a travel time directly inferred from periodic or quasi-periodic data. The present study expands on these innovations and generalizes the approach to arbitrary high-order chains with time-delay coordinates (*21*) enabled by array indexing to model complex and possibly chaotic nonlinear dynamics, and introduces a control-oriented extension to include external inputs and control. Besides its accuracy, one major advantage that the method has is the ability to control the resolution level through adaptive coarse graining.

Dynamics of complex systems is often driven by complicated small-scale (sometimes microscopic) interactions (e.g., turbulence and biological signaling) that are either unknown or very expensive to fully resolve (*22*). The resolution of CNM can be adapted to match any desired level, even when microscopic details are not known. This universal representation of strongly nonlinear dynamics, enabled by adaptive coarse graining and a probabilistic foundation, promises to revolutionize our ability to understand, estimate, predict, and control complex systems in all scientific fields. The method is inherently robust and honest to the data. It requires no assumption on the analytical structure of the model and is computationally tractable, even for high–degree of freedom problems. A code is available at https://github.com/fernexda/cnm.

### Cluster-based network modeling

**Fig. 1:**
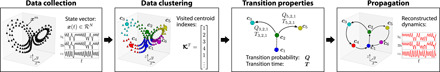
CNM methodology. *M* consecutive *N-*dimensional states ***x***(*t*) ∈ ℛ^*N* × *M*^ are collected at fixed sampling frequency. On the basis of their similarity, the states are grouped into *K* clusters. The network nodes are computed as the cluster centroids ***c****_i_*, and the transition time ***T*** and transition probability ***Q*** between the nodes are identified from the data. The CNM dynamics are propagated as consecutive flights between centroids. Each transition is characterized by its destination, given by ***Q***, and its transit time, given by ***T***.

Robust probability-based data-driven dynamical modeling for complex nonlinear systems has the potential to revolutionize our ability to predict and control these systems. Cluster-based network models reproduce the dynamics on a directed network, where the nodes are the coarse-grained states of the system. The transition properties between the nodes are based on high-order direct transition probabilities identified from the data. The model methodology is applied to a variety of dynamical systems, from canonical problems such as the Lorenz attractor to rare events to high–degree of freedom systems such as a boundary layer flow simulation. The general methodology is illustrated in Fig. 1 with the Lorenz system and is detailed in the following.

#### Data collection and clustering

The starting point of CNM is the data collection of *M* consecutive discrete *N-*dimensional state of the system ***x***(*t*) ∈ ℛ^*N*^ equally spaced in time with Δ*t* such that the state at *t^m^* is $x(t^{m})=x(m\Delta t)=[x_{1}^{m},\ldots ,x_{N}^{m}]$. The state ***x*** can consist of the full state, a low-dimensional representation of the full state, or an observable. The discrete states are grouped into *K* clusters C*_k_*, and the network nodes are identified as the clusters’ centroids ***c****_k_*, defined as the average of the states in each cluster. In this study, clustering is achieved with the unsupervised *k*-means++ algorithm (*23*, *24*) that minimizes the inner-cluster variance. In other words, the algorithm organizes the data such that the inner-cluster similarity is maximized and the intercluster similarity is minimized. Other clustering algorithms are possible. The choice is a problem-dependent option. The vector $K=[K_{1},\ldots ,K_{I}]$, K*_i_* ∈ [1, *K*], contains the indexes of the consecutively visited clusters over the entire time sequence such that K*_i_* is the index of the *i*th visited cluster. The first and last clusters are C_K_1__ and C_K*_I_*_, respectively. The size *I* of K is equal to the number of transitions between *K* centroids over the entire ensemble plus one. We note that two sequential cluster visits are not necessarily equally spaced in time but rather depend on the state’s rate of change in their vicinity.

#### Transition properties

Before we detail the transition properties of cluster-based network models (*20*), we briefly review those of cluster-based Markov models (*19*) upon which the current method builds. In cluster-based Markov models, the state variable is the cluster population **q** = [*q*_1_, …, *q_K_*]^T^, where *q_k_* represents the probability to be in cluster *k* and the superscript T denotes the transpose. The transitions between clusters are modeled with a first-order Markov model. The probability to move from cluster C*_j_* to cluster C*_k_* is described by the transition matrix **P** = (*P*_*k*, *j*_) ∈ ℛ^*K* × *K*^ as

$$P_{k,j}=\text{Pr}(K_{i}=k|K_{i-1}=j)$$ (1)

The transition matrix **P** is computed as

$$P_{k,j}=\frac{n_{k,j}}{n_{j}}$$ (2)

where *n*_*k*, *j*_ is the number of samples that move from C*_j_* to C*_k_* and *n_j_* is the number of transitions departing from C*_j_* regardless of the destination point.

The transition time Δ*t* is a user-specified constant. Let **q***^l^* be the probability vector at time *t^l^* = *l*Δ*t*; then, the change in one time step is described by

$$q^{l+1}=P\;q^{l}$$ (3)

With time evolution, Eq. 3 converges to the asymptotic probability $q^{\infty }≔\text{lim}\;l\to \infty q^{l}$. In a typical case, Eq. 3 has a single fixed point **q**^∞^.

Conversely, CNM relies on the direct transition matrix **Q**, which ignores inner-cluster residence probability and only considers intercluster transitions. The inner-cluster residence probability refers to that of staying in the same cluster, whereas the intercluster probability refers to that of transitioning to another cluster. The direct transition probability is inferred from data as

$$Q_{k,j}=\frac{n_{k,j}}{n_{j}}$$ (4)

with *Q*_*j*, *j*_ = Pr (K*_i_* = *j*∣K_*i*−1_ = *j*) = 0, by the very definition of a direct transition. We emphasize that despite their similarity, Eqs. 2 and 4 define two different properties. Generalizing to an *L-*order model, which is equivalent to using time-delay coordinates, the direct transition probability is expressed as Pr(K*_i_*∣K_*i* − 1_, …, K_*i*−*L*_). Illustrating for a second-order model, the probability to move to C*_l_* having previously visited C*_k_* and C*_j_* is given by

$$Q_{l,k,j}=\text{Pr}(K_{i}=l|K_{i-1}=k,K_{i-2}=j)$$ (5)

Time-delay embedding is a cornerstone of dynamical systems (*25*). The optimal Markov chain order *L* is problem dependent. Larger *L* values are typically necessary for problems with complex phase-space trajectories. In this study, we shall demonstrate how time-delay embedding benefits extend to higher-order cluster-based network models.

**Fig. 2:**
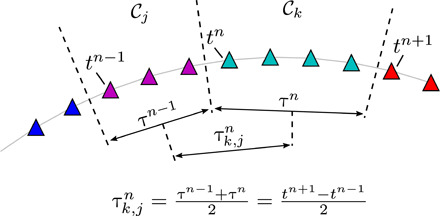
Definition of the transition time from cluster C*_j_* to C*_k_*. The transit time τ*^n^* in C*_k_* at iteration *n* is the time range spanned by the data entry and exit times in the clusters, *t^n^* and *t*^*n* + 1^. The individual transition time $\tau_{k,j}^{n}$ is defined as the average transit time between two neighboring clusters.

The second transition property is the transition time. For Markov models, the time step is a critical user-defined design parameter. If the time step is too small, then the cluster-based Markov model idles many times in each cluster for a stochastic number of times before transitioning to the next cluster. The model-based transition time may thus substantially deviate from the deterministic data-driven trajectories through the clusters. If the time step is too large, then one may miss intermediate clusters. This design parameter can be avoided in CNM. The key idea is to abandon the “stroboscopic” view and focus on nontrivial transitions, thus avoiding rapid state diffusion to a fixed point representing the posttransient attractor. Let *t^n^* and *t*^*n* + 1^ be the time of the first and last snapshots to enter and, respectively, to leave C*_k_* at the *n*th iteration (Fig. 2). Here, iterations refer to the sequential jumps between the centroids. The residence time τ*^n^* = *t*^*n* + 1^ − *t^n^* corresponds to the duration of the state transit in cluster C*_k_* at this iteration. We define the individual transition time from cluster *j* to cluster *k* for one iteration as half the residence time of both clusters

$$\tau_{k,j}^{n}=\frac{\tau^{n-1}+\tau^{n}}{2}=\frac{t^{n+1}-t^{n-1}}{2}$$ (6)

Averaging all *n*_*k*, *j*_ individual transition times from cluster C*_j_* to C*_k_* yields the transition time $T_{k,j}=1/n_{k,j}\sum_{n=1}^{n_{k,j}}\tau_{k,j}^{n}$. This definition may appear arbitrary but is the least-biased guess consistent with the available data. Similar to the direct transition matrix **Q** for an *L*-order chain, the transition time matrix **T** = (*T*_*k*, *j*_) ∈ ℛ^*K* × *K*^ also depends on the *L* − 1 previously visited centroids. When *L* is large, this could yield to two storage-intensive *L* + 1−dimensional tensors **Q** and **T** with *K*^*L* + 1^ elements. The expensive tensor creation and storage is circumvented by a lookup table, where only nonzero entries that correspond to actual transitions are retained. The lookup tables are typically orders-of-magnitude smaller than the full tensors (see section S1).

#### Propagation

The final step in CNM propagates the state motion. We assume a uniform state propagation between two centroids ***c****_j_* and ***c****_k_* as

$$x(t)=\alpha_{\mathrm{kj}}(t)c_{k}+[1-\alpha_{\mathrm{kj}}(t)]c_{j},\;\alpha_{\mathrm{kj}}=\frac{t-t_{j}}{T_{k,j}}$$ (7)

where *t_j_* is the time when the centroids ***c****_j_* is left. The motion between the centroids may be interpolated with splines for smoother trajectories. As CNM is purely data driven, the model quality is directly related to that of the training data. More specifically, the sampling frequency and total time range must be selected such that all relevant dynamics are captured and are statistically fully converged. This usually requires a larger amount of data than other data-driven methods, such as ARMA and SINDy.

## RESULTS

### CNM of the Lorenz system

**Fig. 3:**
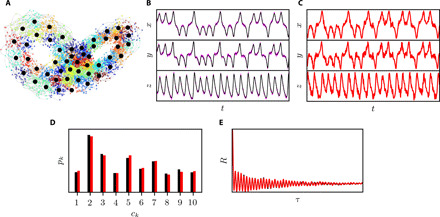
CNM of the Lorenz system with 10% uniformly distributed superimposed stochastic noise. (**A**) Phase-space representation of the data clustering. The centroids are depicted with black circles, and the small circles are the snapshots, colored by their cluster affiliation. The CNM accuracy is demonstrated in the accurate reproduction of (**B** and **C**) the time series, (**D**) the CPD, and (**E**) the autocorrelation function. Black, purple, and red colors denote the reference clean, the reference noisy, and CNM data, respectively.

CNM is applied to the Lorenz system, a widely used canonical chaotic dynamical system (*26*) defined by three coupled nonlinear differential equations

$$\begin{array}{c} \frac{\mathrm{dx}}{\mathrm{dt}}=\sigma (y-x)\\ \frac{\mathrm{dy}}{\mathrm{dt}}=x(\rho -z)-y\\ \frac{\mathrm{dz}}{\mathrm{dt}}=\mathrm{xy}-\beta z \end{array}$$ (8)

where the system parameters are here defined as σ = 10, ρ = 28, and β = 8/3. To assess the method’s robustness, the dynamical system is superimposed with a uniformly distributed stochastic noise with an amplitude of 10% of that of the clean signal. The data are clustered with *K* = 50 centroids, depicted in Fig. 3A. The snapshots are colored on the basis of their cluster affiliations. CNM is performed with a chain order *L* = 22 using ≈17,000 transitions, which cover the same time range as that of the original data. The optimal *K* and *L* values are problem dependent. They are identified for the Lorenz system through a parametric study, where the root mean square error of the autocorrelation function between the reference data and the model is minimized. The autocorrelation computation is described in section S2. Suboptimal *K* and *L* values evidently degrade the model performance in a case-dependent manner. Typically, the number of clusters is related to the desired level of resolution and the level of complexity in the dynamics. Too few centroids might oversimplify the dynamics, whereas too many might lead to a noisy solution. The chain order *L* is strictly dictated by the complexity of the dynamics in the phase space. A system with a highly irregular trajectory with multiple intersections typically requires higher chain order. A detailed analysis of the model hyperparameter selection and error topology is presented in section S3.

Time series obtained with CNM agree very well with the reference data (Fig. 3, B and C). We note that the black and purple colors in Fig. 3B denote the reference clean and noisy data, respectively. The oscillating amplitude growth in both ears, as well as the ear switching, is correctly captured by the model. Inherent to the method, the noise in the training data is mirrored in the reconstructed CNM time series. The model remains faithful to the training data. It might be, however, described as too faithful, as it also reproduces the measurement noise in the model dynamics. CNM cannot disambiguate between true dynamics and noise. This shortcoming is also the method’s strength, as the model remains robust regardless of the noise level (up to 70% noise level is tested). A detailed analysis of the noise influence on CNM is provided in section S7, where noise levels of up to 70% are superimposed on the Lorenz system and their effects on the dynamics were analyzed.

The cluster probability distribution (CPD) *p_k_*, *k* = 1, …, *K*, provides the probability of the state to be in a specific cluster. It indicates whether the modeled trajectories populate the phase space similarly to the reference data (see section S2). The CPD for both the clean data and CNM is shown in Fig. 3D. We purposely show the CPD of the clean data to assess deviation from the original system. For clarity, *p_k_* is shown with 10 clusters only instead of the full 50 clusters. As the figure shows, CNM accurately reproduces the probability distribution. Following Protas *et al.* (*27*), the cluster-based network model is validated on the basis of the autocorrelation function of the state vector. This function avoids the problem of comparing two trajectories with finite dynamic prediction horizons due to phase mismatch. The autocorrelation function also yields the fluctuation energy (or variance) at zero time lag *R*(0) and can be used to infer the spectral behavior. As Fig. 3E shows, CNM accurately reproduces the fast oscillatory decay, even after dozens of oscillations, as well as the fluctuation energy *R*(0), which is reproduced with a 2.8% root mean square error. This performance is in contrast to the cluster-based Markov models, where time integration leads to the average flow, and to first-order cluster-based network models (*20*), where the prediction accuracy is much lower. A detailed comparison between the cluster-based Markov model, the first-order cluster-based network model, and the current model is provided in section S4.

### Demonstration on examples

**Fig. 4:**
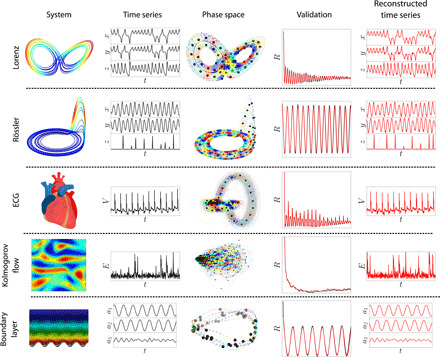
The CNM implemented on five applications covering a wide range of dynamics. The first two applications are three-dimensional chaotic systems, the Lorenz and Rössler attractors. The two following examples are one-dimensional experimental measurements from an ECG and numerical simulation of the dissipation energy in a Kolmogorov flow. The final application is a large eddy simulation of an actuated turbulent boundary layer. The excellent match of the autocorrelation functions for all applications demonstrates the CNM’s ability to capture the relevant dynamics for any complex nonlinear system. The modeled time series faithfully reconstruct the data including the intermittent quasi-random bursts of the Kolmogorov dissipation energy, as well as the *z-*component pulses of the Rössler system.

CNM is applied to numerous examples, ranging from analytical systems to real-life problems using experimental and simulation data. The main results are summarized in Fig. 4. Details on each application are provided in Materials and Methods. The first two applications are the Lorenz (*26*) and Rössler (*28*) attractors, typical candidates for dynamical systems analysis. The two systems are governed by simple equations and exhibit chaotic behavior under specific parameter values. The following two implementations are one-dimensional systems: electrocardiogram (ECG) measurements (*29*) and the dissipative energy from a Kolmogorov flow (*30*). Whereas the ECG exhibits the regular heartbeat pattern, the dissipative energy of the Kolmogorov flow is quasi-random with intermittent bursts. The last CNM application is a high-dimensional large eddy simulation of an actuated turbulent boundary layer for skin friction reduction (*31*). The clustering step on this ≈5 million grid cell simulation is performed on the mode coefficients of a lossless POD. We note that other dimensionality reduction techniques than POD are also possible. This dimensionality reduction step substantially reduces the computational load while yielding the same clustering outcome as the full difference matrix (*19*). The boundary layer time series are therefore represented with the mode coefficients.

In each example, both the qualitative and quantitative dynamics are faithfully captured. The reconstructed time series are hardly distinguishable from the original data. Intermittent events such as the peaks in the Rössler *z* component and the dissipation energy bursts of the Kolmogorov flow are statistically very well reproduced. The autocorrelation distributions of both reference data and models match accurately over the entire range, demonstrating both robustness and accuracy. We note that robustness is inherent to CNM, because the modeled state always remains close to the training data.

**Fig. 5:**
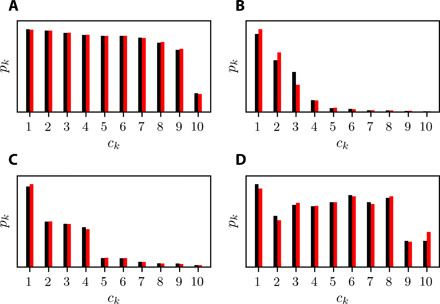
CPD of the data and CNM for four applications. (**A** to **D**) CPD of the Rössler system, ECG signal, Kolmogorov flow dissipation energy, and actuated turbulent boundary layer, respectively. For all cases, the data (black) and CNM (red) are in good agreement. The specific features of each dataset, such as the rare events of the Kolmogorov dissipation energy and the fast heartbeat pulses, are probabilistically well reconstructed by CNM.

The CPD of the data and CNM for the Rössler system, the ECG signal, the Kolmogorov flow dissipation energy, and the actuated turbulent boundary layer are presented in Fig. 5. For all cases, CNM accurately reconstructs the distributions. The probabilities of less visited clusters corresponding to fast events such as the peaks in the *z* directions of the Rössler attractor (Fig. 5A) and the heartbeat pulse (Fig. 5B) or to rare events for the Kolmogorov flow (Fig. 5C) are very well captured by CNM. We note that a low cluster probability is only a postprocessing step to identify rare events. Details about CNM’s ability to predict a rare event ahead of time are provided below.

**Fig. 6:**
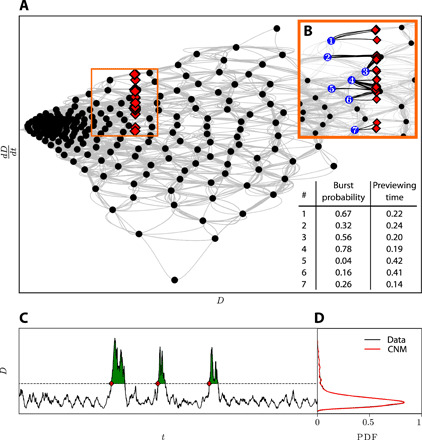
Rare events from the Kolmogorov flow dissipation energy. (**A**) The phase space spanned by the dissipation energy *D* and its time derivative $\overset{\cdot }{D}$ constructed using CNM. Snapshots delimiting the onset of bursts are marked by the red diamonds and are concentrated in a specific region in the phase space. A close-up of the phase space around the red diamonds is shown in (**B**), where the last visited clusters preceding a burst are marked in blue. The table on the bottom right of (A) lists the corresponding burst probability and the prediction time at the seven blue centroids. A portion of the dissipation time series is presented in (**C**). The dashed line denotes an arbitrary threshold beyond which the peaks, represented with green filling, are considered a burst. (**D**) Probability distribution of the data (black) and CNM (red). Both the main peak and the decaying tail of the distribution are accurately reproduced.

A special characteristic of CNM is its ability to accurately model and predict systems with rare events. This ability is rooted in the probabilistic framework upon which CNM is constructed, where the recurrence properties are the same as the reference data. If one cluster is visited multiple times (or seldom) in the data, it will also be a recurrence point of the CNM. A generic example of a rare event problem is the Kolmogorov flow (*32*), a two-dimensional incompressible flow with sinusoidal forcing. With a sufficiently high forcing wave number, the flow becomes unstable and the dissipation energy *D* exhibits intermittent and spontaneous bursts (see Fig. 6C). The dashed line denotes an arbitrary threshold beyond which a peak is considered a rare event. The probability distribution function (PDF) of the dissipation energy from the data and CNM is compared in Fig. 6D.

The main peak centered around zero reflects the stochastic nature of the dissipation energy, whereas the tail depicts rare events whose occurrence probability decreases with their amplitude. As the figure shows, CNM accurately captures the probabilistic behavior of the dissipation energy. Both the main stochastic peak and the rare event tail of the distribution are well reconstructed. Moreover, the total number of bursts in the current sequence is well reproduced, with 58 bursts in the original data compared to 62 for CNM.

Besides reproducing the dynamics, CNM offers powerful capabilities to predict and thus control rare events. Figure 6A presents the phase space spanned by the dissipation energy *D* and its time derivative $\overset{\cdot }{D}$ constructed using CNM. Snapshots delimiting the onset of bursts are marked by the red diamonds and are concentrated in a specific region in the phase space. Dynamics crossing the red diamonds from the left will experience a burst on the right before returning to the left region from below. A close-up of the phase space around the red diamonds is shown in Fig. 6B, where the last visited clusters preceding a burst are marked in blue. The table on the bottom right of Fig. 6A lists the corresponding burst probability and the previewing time at the seven blue centroids. The burst probability represents the probability to encounter a burst during the next motion propagation from the current centroid. High burst probabilities mark a high likelihood to encounter a burst. The previewing time denotes the look-ahead time from the centroid to the burst onset. In practice, a limit on the burst probability can be selected, above which an action (e.g., control) with a certain previewing time to execute can be taken. For the settings used (*K* = 200, *L* = 25), the burst probabilities and the previewing times at the seven listed centroids range between 0.04 and 0.78% and between 0.14 and 0.42 time units, respectively. The burst probability and previewing time at other centroids away from this region are negligibly low.

### Control-oriented CNM

**Fig. 7:**
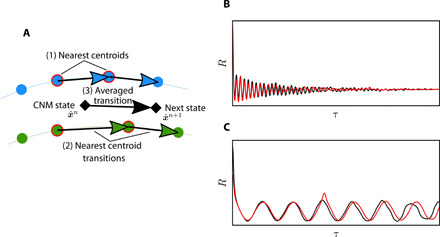
Control-oriented CNM. (**A**) CNMc iteratively propagates the state in the phase space populated with the centroids from the two operating conditions with the closest control parameters. (1) Neighboring centroids to the current state ${\hat{x}}^{n}$ at iteration *n* are first identified. (2) Their transition properties are calculated and then (3) averaged to determine the next state ${\hat{x}}^{n+1}$. CNMc accuracy is demonstrated by the autocorrelation function distributions of the data (black) and the predicted case (red) for the (**B**) Lorenz system and the (**C**) actuated turbulent boundary layer, respectively.

To disambiguate the effect of internal dynamics from actuation or external input, we generalize CNM to include control ***b***. We note that the current control-oriented CNM (CNMc) version is only suitable for autonomous forcing, where the input ***b*** is constant and time independent. The transition probabilities ***Q***(***b***) and transition times ***T***(***b***) are first identified for each actuation setting ***b*** individually. The three-step procedure for the propagation of a new control command $\hat{b}$ depicted in Fig. 7A is then performed. At each iteration, (i) a search for the nearest centroids from the two closest actuation test cases is performed. (ii) Their transition properties are then identified and (iii) averaged to determine the transition of the state $\hat{x}$. More details of the CNMc algorithm are provided in section S5. CNMc is applied to two systems at new control conditions, the Lorenz attractor and the actuated turbulent boundary layer. The Lorenz system with ρ = 28 is interpolated from two test cases with ρ = 26 and ρ = 30, and the boundary layer with actuation parameters λ^+^ = 1000, *T*^+^ = 120, and *A*^+^ = 30 is interpolated from cases with λ^+^ = 1000, *T*^+^ = 120, and *A*^+^ = 20 and λ^+^ = 1000, *T*^+^ = 120, and *A*^+^ = 40. The CNMc settings are provided in section S6. Despite the algorithm’s simplicity, the main dynamics are properly captured, as shown by the autocorrelation functions in Fig. 7 (B and C) and the time series (fig. S7). CNMc is cast in the same probabilistic framework as CNM and thereby retains all previously demonstrated advantages. As the dynamics are interpolated from centroids that belong to potentially different trajectories, the resulting motion might be noisier and a larger number of centroids than regular CNM are typically required.

## DISCUSSION

We propose a universal data-driven methodology for modeling nonlinear chaotic and deterministic dynamical systems. The method builds on prior work in cluster-based Markov modeling and network dynamics. CNM has several unique and desirable features. (i) It is simple and automatable. Once the various schemes are chosen (e.g., clustering algorithm and transition time), only two parameters must be selected: the number of clusters *K* and the Markov chain order *L*. Too few centroids might oversimplify the dynamics, whereas too many might lead to a noisy solution. We note that a high Markov chain order *L* is not always necessarily advantageous. Both parameters are problem dependent and can be automatically optimized. (ii) The method does not require any assumption on the analytical structure of the model. It is always honest to the data. (iii) The offline computational load is low. The most expensive step in the process is the occasionally required snapshot-based POD for dimensionality reduction. After the POD computation, the clustering and network modeling require a tiny fraction of the computational operation. Details on the algorithm computational load are provided in section S6. (iv) The recurrence properties are the same as the reference data. If one cluster is visited multiple times (or seldom) in the data, it will also be a recurrence point of the CNM. This feature is what enables modeling of problems with rare events. (v) Long-term integration will never lead to divergence, unlike, e.g., POD-based models. The simplicity and robustness, however, have a price. On the kinematic side, the simple CNM version cannot extrapolate, e.g., resolve oscillations at higher amplitudes not contained in the data. On the dynamic side, we lose the relationship to first principles: The network model is purely inferred from data, without links to the governing equations. In particular, cluster-based models are not natural frameworks for dynamic instabilities, as the notion of exponential growth and nonlinear saturation is intimately tied to Galerkin expansions. Subsequent generalizations need to overcome these restrictions. (vi) The framework is generalizable, allowing control-oriented predictions beyond the training data. A simple interpolation-based control-oriented extension of CNM is proposed and tested. Despite its simplicity, CNMc accurately predicts the state dynamics at new operating conditions over the entire sample record.

CNM is found to have a distinct superiority over cluster-based Markov models, namely, the much longer prediction horizon as evidenced by the autocorrelation function. The modeling and prediction capabilities are demonstrated on a number of examples exhibiting chaos, rare events, and high dimensionality. In all cases, the dynamics are remarkably well represented with CNM; the temporal evolution of the main flow dynamics, the fluctuation level, the autocorrelation function, and the cluster population are all accurately reproduced. In a computational fluid dynamics analogy, the cluster-based Markov models may be compared with unsteady Reynolds-averaged Navier-Stokes equations describing the transient mean flow and the CNM with large eddy simulations describing the coherent structures.

CNM opens a novel automatable avenue for data-driven nonlinear dynamical modeling and real-time control. It represents a new powerful tool in the existing large toolbox of dynamical system identification and reduced-order modeling. It holds the potential for a myriad of further research directions. Its probabilistic foundations are naturally extendable to include uncertainty quantification and propagation. One limiting requirement of CNM is the relatively large statistically converged training data that it requires compared to other known methods (e.g., ARMA and SINDy). This requirement could be relaxed through explicit coupling to first-principle equations. The control-oriented extension may be further refined and more broadly implemented on other applications.

## MATERIALS AND METHODS

In this section, we detail the various systems including the numerical setup and the CNM modeling parameters. CNM is fully parametrized by the number of clusters *K* and the model order *L*. Their selection plays an important role in the model accuracy. The values used for the various systems are listed in Table 1. The procedure to select *K* and *L* is detailed in section S3. The last column in Table 1 lists the normalized time delays *t_L_*/*T*_0_, where *T*_0_ is the fundamental period computed from the dominant frequency identified from the autocorrelation function. For purely random signals with no deterministic component, such as the dissipative energy of the Kolmogorov flow, no characteristic period can be defined.

**Table 1:** CNM settings for all applications. The number of clusters *K* and the model order *L* are listed for the five systems. The last column *t_L_*/*T*_0_ designates the normalized time delay corresponding to the selected model order *L*. The fundamental period *T*_0_ is computed from the dominant frequency of the system, when possible.

**System**	**Number of** **clusters *K***	**Model order *L***	***t_L_*/*T*_0_**
Lorenz	50	22	1.7
Rössler	100	2	0.6
ECG	50	23	0.14
Kolmogorov flow	200	25	–
Boundary layer	50	3	0.25

As indicated by the table, the CNM parameters are strongly dependent on the nature of the systems dynamics. Physical interpretation of the chosen parameters is provided for each system in the following.

### Lorenz system

The Lorenz system (*26*) is a typical candidate for dynamical system analysis. Despite its low dimension, it exhibits a chaotic behavior. The motion is characterized by periodic oscillations of growing amplitude in the “ears” and a random switching between them. The Lorenz system is driven by a set of three coupled nonlinear ordinary differential equations given by

$$\begin{array}{c} \frac{\mathrm{dx}}{\mathrm{dt}}=\sigma (y-x)\\ \frac{\mathrm{dy}}{\mathrm{dt}}=x(\rho -z)-y\\ \frac{\mathrm{dz}}{\mathrm{dt}}=\mathrm{xy}-\mathrm{bz} \end{array}$$ (9)

The selected parameters are σ = 10, ρ = 28, and β = 8/3 with initial conditions ( − 3,0,31). The simulation is performed with a time step Δ*t* = 0.015 for a total of 57,000 samples. The numerical integration is performed with the explicit Runge-Kutta method of fifth order using the SciPy library from the Python programming language (*33*, *34*).

The relatively high number of clusters (*K* = 50) ensures that each wing is resolved by two orbits of centroids (see the phase-space clustering in Fig. 4) and allows us to reproduce some of the increasing oscillation amplitude. *K* can be increased (decreased) to resolve more (less) orbits in each ear. Because of the dynamic complexity and especially the random ear flipping, the Lorenz system requires a large time delay *t_L_* equivalent to 1.7 rotations. With lower *L* values, the trajectory that reaches the ear intersection becomes more likely to wrongly switch sides.

### Rössler system

The Rössler is a three-dimensional system governed by nonlinear ordinary differential equations (*28*) that read

$$\begin{array}{c} \frac{\mathrm{dx}}{\mathrm{dt}}=-y-z\\ \frac{\mathrm{dy}}{\mathrm{dt}}=x+\text{ay}\\ \frac{\mathrm{dz}}{\mathrm{dt}}=b+z(x-c) \end{array}$$ (10)

where the parameters are *a* = 0.1, *b* = 0.1, and *c* = 14. The initial conditions are set to (1,1,1), and the simulation is performed with a time step Δ*t* = 0.01 for a total of 50,000 samples. The Rössler data are also created with the SciPy library using the explicit Runge-Kutta method of fifth order. Similar to the Lorenz system, the Rössler is widely used for dynamical system analysis. The system also yields chaotic behavior under specific parameter combinations. The motion is characterized by rotations of slowly growing amplitude in the *x-y* plane and intermittent peaks in the *z* direction.

The Rössler system requires a large number of clusters to ensure a sufficient centroid coverage in the peak for an accurate reproduction of this intermittent and fast event. However, because the trajectory itself is relatively simple, a time delay *t_L_* of approximately half of the characteristic period is sufficient (*t_L_*/*T*_0_ = 0.6).

### ECG signal

An ECG measures the heart activity over time. Electrodes are placed on the person’s skin to deliver a univariate voltage of the cardiac muscle movements. The time series exhibit the typical pulse associated with the heartbeat. The ECG signal used in this study is from the PhysioNet database (*35*). The signal time range is 180 s, and the sampling frequency is 250 Hz.

Similarly to the Rössler, the ECG requires a large number of clusters *K* to resolve the quasi-circular phase-space trajectory corresponding to the fast heartbeat pulse. Again, because of the very regular and repetitive nature of the heart activity, a small time delay *t_L_* is sufficient.

### Kolmogorov flow

The Kolmogorov flow is a two-dimensional generic flow defined on a square domain ***q*** = (*x*, *y*) with 0 ≤ *x* ≤ *L* and 0 ≤ *y* ≤ *L*, subject to a horizontal sinusoidal forcing ***f***, defined by

$$f(x)=\text{sin}\;(a\;y)e_{1}$$ (11)

where ***e***_1_ = (1,0)*^T^* is a unit vector in the *x* direction. The Kolmogorov flow is a test bed for various fluid mechanics and turbulence studies (*36*). The temporal evolution of the flow energy *E*, the dissipative energy *D*, and input energy *I* are defined by

$$E(t)=\frac{1}{2L^{2}}\iint {|u(q,t)|}^{2}dq$$ (12)

$$D(t)=\nu \frac{1}{L^{2}}\iint {|\omega (q,t)|}^{2}dq$$ (13)

$$I(t)=\frac{1}{L^{2}}\iint {|u(q,t)\cdot f(q,t)|}^{2}dq$$ (14)

where ν is the fluid viscosity and ω is the vorticity. The rate of change of the energy is equal to the input energy minus the dissipation energy, as $\overset{\cdot }{E}=I-D$. With increasing forcing wave number *a*, the dissipation energy yields intermittent and random bursts. This behavior makes the dissipation energy a good candidate for rare event modeling. The current data were created and shared by Farazmand and Sapsis (*37*), with a wavenumber *a* = 4 and a Reynolds number *Re* = 40. The total time range is 100,000 dimensionless time units with a sampling frequency of 10.

The trajectory in the phase space spanned by *D* and its temporal derivative $\overset{\cdot }{D}$ (Fig. 4) is particularly complex. The region with higher cluster density in the left region of the phase space corresponds to the random fluctuations, and the region with sparser centroid distribution describes the intermittent energy bursts. Because of its stochastic nature and the absence of deterministic patterns, the Kolmogorov flow dissipation energy has been particularly challenging to model. With sufficiently large *K* and *L*, CNM is capable of modeling *D* with high accuracy.

### Actuated turbulent boundary layer

The reduction of viscous drag is crucial for many flow-related applications such as airplanes and pipelines, as it is a major contributor to the total drag. Many passive (*38*, *39*) and active (*40*, *41*) actuation techniques have been investigated to reduce the skin friction drag. In this study, skin friction reduction on a turbulent boundary layer is achieved by means of a spanwise traveling surface wave (*31*, *42*).

The waves are defined by their wavelength λ^+^, period *T*^+^, and amplitude *A*^+^. The superscript + denotes variables scaled with the friction velocity and the viscosity. Details about the computational setup can be found in the work of Albers *et al.* (*31*). The actuation parameters are λ^+^ = 1000, *T*^+^ = 120, and *A*^+^ = 60. The total time range in ^+^ units is 846, and the sampling frequency is 0.5, resulting in 420 snapshots. The velocity field is given by ***u***(***q***, *t*^+^), where ***q*** = (*x*^+^, *y*^+^, *z*^+^) in the Cartesian coordinates with *x*^+^ ∈ [2309,4619], *y*^+^ ∈ [0,692], and *z*^+^ ∈ [0,1000].

Clustering of large high-dimensional datasets is costly. The required distance computation between two snapshots ***u****^m^* and ***u****^n^*

$$d(u^{m},u^{n})={\left\|u^{m}-u^{n}\right\|}_{\Omega }$$ (15)

is computationally very expensive. Here, the norm is defined as

$$∥u{∥}_{\Omega }=\sqrt{{\left(u,u\right)}_{\Omega }}$$ (16)

and the inner product in the Hilbert space ℒ(Ω) of square-integrable vector fields in the domain Ω is given by

$${\left(u,v\right)}_{\Omega }=\int_{\Omega }u(q)v(q)\;dq$$ (17)

For high-dimensional data such as the boundary layer velocity field, data compression with lossless POD can reduce the computational cost of clustering. Here, a snapshot ***u****^m^* is exactly expressed by the POD expansions as

$$u(q,t)=u_{0}(q)+\sum_{i=0}^{M-1}a_{i}(t)\Phi_{i}(q)$$ (18)

where ***u***_0_ is the mean flow, Φ*_i_* denotes the POD modes, and *a_i_*(*t*) are the corresponding mode coefficients. As shown by Kaiser *et al.* (*19*), the distance computation (Eq. 15) can be alternatively performed with the mode coefficients instead of the snapshots, as

$$d(u^{m},u^{n})={\left\|u^{m}-u^{n}\right\|}_{\Omega }$$ (19)

$$=∥a^{m}-a^{n}∥$$ (20)

Hence, $a^{m}=[a_{1}^{m},\ldots ,a_{M-1}^{m}]$ becomes the POD representation of snapshot *m* at time *t^m^* = *m*Δ*t*. Equation 20 is computationally much lighter than (*19*). Despite the additional autocorrelation matrix computation for the POD process, the data compression procedure remains very beneficial for large numerical grids. According to (*20*), the computational savings amount to

$$\frac{M+1}{2J\times I\times K}$$ (21)

where *M* is the number of snapshots, *K* is the number of clusters, *I* is the number of *k*-means inner iterations, and *J* is the number of random centroid initializations. For typical values (*K* ∼ 10, *I* ∼ 10*K*, and *J* ∼ 100), the savings are one or two orders of magnitude. Furthermore, POD is computed only once for each dataset and will benefit all future clusterings performed on that dataset.

The actuated turbulent boundary layer at the used actuation settings exhibits synchronization with the actuation wave. The dynamics show quasi–limit cycle behavior with superimposed wandering. Therefore, a low number of centroids are sufficient to capture the dynamics. If desired, the limit cycle meandering associated with higher frequency turbulence can be resolved with a larger set of centroids. The selected value of *K* = 50 is a compromise between a sufficient resolution of the turbulence scales (64% of the data fluctuation is resolved) and a reasonable model complexity. The dynamics are well captured with a low model order *L*, equivalent to a time delay of a quarter of the actuation period.

## SUPPLEMENTARY MATERIALS

Supplementary material for this article is available at http://advances.sciencemag.org/cgi/content/full/7/25/eabf5006/DC1
